# Valorization of Starch to Biobased Materials: A Review

**DOI:** 10.3390/polym14112215

**Published:** 2022-05-30

**Authors:** Kehinde James Falua, Anamol Pokharel, Amin Babaei-Ghazvini, Yongfeng Ai, Bishnu Acharya

**Affiliations:** 1 Department of Chemical and Biological Engineering, University of Saskatchewan, 57 Campus Drive, Saskatoon, SK S7N 5A9, Canada; kehinde.falua@usask.ca (K.J.F.); hdk062@usask.ca (A.P.); amin.babaei@usask.ca (A.B.-G.); 2 Department of Agricultural & Biosystems Engineering, University of Ilorin, Ilorin PMB 1515, Nigeria; 3 Department of Food and Bioproduct Sciences, University of Saskatchewan, 51 Campus Drive, Saskatoon, SK S7N 5A8, Canada; yongfeng.ai@usask.ca

**Keywords:** starch-based aerogels, carbohydrate polymers, nanofillers, ScCO_2_ drying

## Abstract

Many concerns are being expressed about the biodegradability, biocompatibility, and long-term viability of polymer-based substances. This prompted the quest for an alternative source of material that could be utilized for various purposes. Starch is widely used as a thickener, emulsifier, and binder in many food and non-food sectors, but research focuses on increasing its application beyond these areas. Due to its biodegradability, low cost, renewability, and abundance, starch is considered a “green path” raw material for generating porous substances such as aerogels, biofoams, and bioplastics, which have sparked an academic interest. Existing research has focused on strategies for developing biomaterials from organic polymers (e.g., cellulose), but there has been little research on its polysaccharide counterpart (starch). This review paper highlighted the structure of starch, the context of amylose and amylopectin, and the extraction and modification of starch with their processes and limitations. Moreover, this paper describes nanofillers, intelligent pH-sensitive films, biofoams, aerogels of various types, bioplastics, and their precursors, including drying and manufacturing. The perspectives reveal the great potential of starch-based biomaterials in food, pharmaceuticals, biomedicine, and non-food applications.

## 1. Introduction

One of the standard methods suggested as a lasting solution to the adverse setback of non-biodegradable materials (NBMs) is the use of plant-based materials (PBMs) from compostable sources, especially those of plant origin [1,2]. PBMs contain many valuable substances such as carbohydrates, amino acids, lipids, alkaloids, and terpenoids [3]. They emerge as a promising raw material for several products used in major applications such as biopolymer conversion, medicines, lubricants, solvents, surfactants, and other notable chemicals [4]. Meanwhile, little is known about the inherent characteristics of PBM, as some reports emphasize the possibility of toxic substances during conversion processes [5,6]. As such, features such as biodegradability, sustainability, and biocompatibility of materials used in circular economy loops become imperative to eliminate problems associated with their synthetic counterparts. Therefore, significant reasons for considering biopolymers, such as starch, as an alternative lie in their resource renewability [7], abundance [8], biodegradability [7], biocompatibility [9], low-cost [10], sustainability, and flexibility [11]. Given the particular benefits of the natural biopolymers from different starch sources, their application is considered a novel and alternative material compared to petroleum-based polymers. The increasing demand for starch by pharmaceuticals and food processing industries towards natural biopolymers is appealing [12]. However, when considering the economic worth of starch, they have a relatively low market price. Nevertheless, their usefulness cannot be underestimated; they significantly benefit from industries. Therefore, the importance of developing rarely utilized starches into value-added products such as biomaterials (e.g., aerogels and biofoams) with the potential for various applications is currently being researched.

Starch has been used in areas where low density, high surface area, and porosity are of the utmost importance [13,14]. To this end, the development of novel materials such as aerogels and biofoams from starch has increased over the past decades. Besides, extant studies have documented the application of biomaterials for thermal insulation [2,15,16], medical purposes [15,16], chemical additives [17], and food packaging [18,19,20,21,22], amongst many other purposes. Few scholars have researched the pathways of valorizing starch into various products, which are not limited to those manufactured in cosmetics, nutraceuticals, food, and feed additives [23,24]. According to the work of Jogi and Bhat, starch, cellulose, and gluten are potential raw materials for biobased plastics. The authors further affirmed that the combination of natural organic sources such as starch and gelatin is also one of the most efficient bioplastics methods [12]. Furthermore, attention is increasing towards the use of starch-rich biomass as a novel product for bioplastics [25,26]. It can be deduced from numerous pieces of literature that starch could be valorized into a variety of novel products, but bioplastics are more prominent than porous materials such as foams and aerogels. Evidently, starch as a byproduct material is now becoming competitive in the field of biopolymers. The increasing demand for porous materials is a point of interest all over the world. For instance, the Asia-Pacific region leads biofoam packaging, closely followed by America, including the United States, Canada, and Mexico [27]. From an economic viewpoint, the aerogel market is expected to grow at a compound annual growth rate (CAGR) of 30.8% to 31% from 2017 to 2023, while the biofoam packaging market is expected to grow at a 5.50% CAGR from 2020 to 2027 [28].

In this paper, we comprehensively present the valorization of starch to biobased materials. The method we adopted was to divide our findings into two sections: the first was to present information about starch, including its history, structure, extraction, and modification, and the second was to emphasize the unique applications of starch. A more in-depth look into the application of starch-based sources in nanofillers, smart/intelligent packaging films, aerogels, and biofoams, and their manufacturing techniques, was offered. The literatures considered in this review were indexed, with a preference for those published within the past five years. However, a few older studies that are important for this study were also incorporated. Finally, research gaps were identified and highlighted, and the outlook in the forthcoming years was also presented.

## 2. Structure of Starch

### 2.1. Amylose

Amylose makes up 5–35% of most natural starches and significantly impacts starch characteristics [29]. Amylose content can range from less than 1% in waxy starches to more than 70% in high amylose starches [30,31], and they are made up of a linear chain of 500–2000 glucose units [32]. Although data have revealed that amylose has several branch linkages, it acts as a linear polymer with linked repeating units in a single flexible chain [33]. Amylose-rich starches have better thermal stability and could produce a more stable amylose-lipid complex. These thermal properties and gel formation are influenced by the physical interaction [34]. More so, amylose is distinct from other sugars in that it can crystallize independently or in the presence of complexing agents [33,35]. The starch pasting and gel properties of a substance are significantly affected by the level of amylose content [36,37]. On a commercial scale, food and industrial applications could benefit considerably from variations in the amylose content of starch [29]. Low amylose starches in cooked foods provide stickier textures than regular starches and form clear, stable pastes appropriate for a wide range of industrial applications [38,39].

### 2.2. Amylopectin

**Figure 1:**
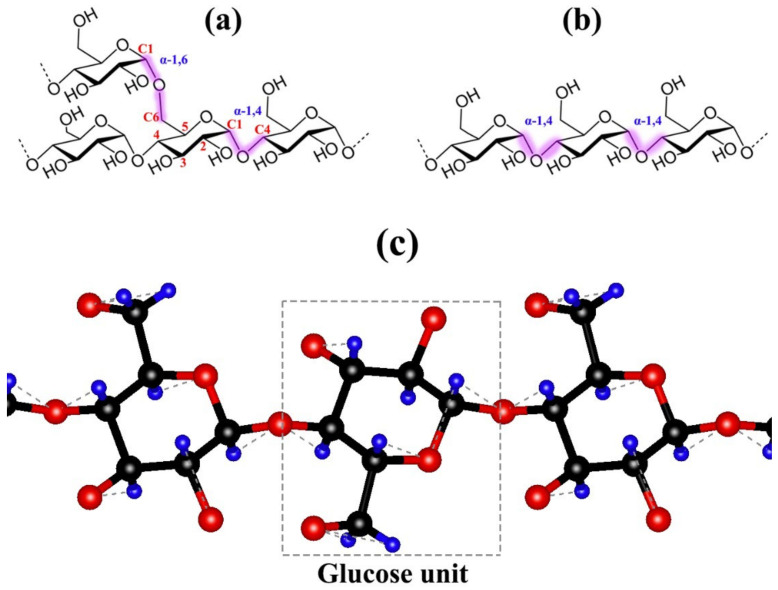
Structure of (**a**) amylopectin, (**b**) amylose, and (**c**) simple starch.

Amylopectin, a component of starch (70–80%) [34], is one of nature’s most enormous molecules due to its substantial glucose units and vital functional characteristics. It has a more complicated molecular structure than amylose and comprises around significantly branched 1,000,000 glucose units [32]. One of the essential elements in the amylopectin chain length distribution is the starch characteristics, which greatly vary depending on the botanical source [30]. The unit chains of amylopectin are divided into three categories: A, B, and C, with each chain having its functions and features [30]. The A chains are the shortest, with a single α-(1 → 6) link to the amylopectin molecule. The B chains are based on length and cluster and are separated into four levels: B1, B2, B3, and B4. B1 chains are part of one cluster, while B2 and B3 chains are two or three clusters, respectively [30]. Chains A and B1 have similarities that affect their pasting properties [34]. However, chains B2-B4 are exclusively considered linking chains in amylopectin molecules [40]. The C chains are a mix of the A and B chains [41], and they serve as the amylopectin molecules’ mainframe support but are constrained by their swelling qualities [41]. In other words, the A chain has its reducing end attached to another B or C chain but does not carry any other chains; the B chain, which not only has its reducing end attached to another B or C chain but also carries other A or B chains; and the C chain, which is the molecule’s only chain with a reducing end. With respect to the degree of polymerization, the A chain has the lowest DP (6–12), while B1–B3 chains have a DP range between 13 and 37, but they could extend further [42]. The C chain in amylopectin preparations has a size distribution that ranges from the degree of polymerization (DP) of 15 to 120, with a peak at a DP of 40. Because a single amylopectin molecule only has one C chain, the length of the C chain varies substantially between individual molecules [42,43]. Compared to heavily branched amylopectin chains, short amylopectin chains producing crystalline clusters have acquired general recognition [33]. The structures of amylose, amylopectin, and a simple starch are depicted in Figure 1.

Retrogradation, gelatinization, gel rheology [44], solubility, cold swelling, water absorption [45], syneresis, viscosity, tackiness, and shear resistance [32] are functional properties that can be used to determine the structural and molecular distribution of amylose and amylopectin when they are subjected to various processing techniques and applications. In the presence of a specific quantity of moisture, the heat treatment action on amylose and amylopectin results in noticeable alterations to the structure of starch [46,47].

## 3. Extraction of Starch

Proteins, carbohydrates, and lipids, which are potential ingredients for the manufacture of enzymes, gelatin, collagen, and other bioactive compounds, are known to be present in discarded and underutilized by-products [48]. This signifies that the extraction products are the potential raw materials for food additives, functional foods, nutraceuticals, pharmaceuticals, and, more crucially, bio-packaging products [49]. Kringel’s colleagues reviewed starch extraction from roots, tubers, pulses, pseudo-cereals, and other unconventional sources [50]. The most well-known starch sources include cereals, potatoes, and tapioca, which are used for starch extraction and direct consumption [51]. Although they have been relatively used in the past decades, pulse crops are gaining popularity in the agri-food industry due to their potential health advantages, as well as their promising supply of starch and protein [52]. Peas, lentils, and faba bean are the three most common pulse crops used in the food sector to extract protein concentrate/isolate, resulting in an increasing amount of pulse starch as a by-product. They are also used as an alternative to cow milk to provide milk with better nutritional value [53]. Pulses have more protein and fat than roots and tuber starches, as well as smaller starch granules, making starch extraction more challenging [50]. The fractionation of starch could be carried out using two approaches: dry milling (DM) and wet milling (WM). However, few scholars have reported situations where certain steps of either of the two approaches have been combined for the extraction of starch from pulse crops, particularly peas, lentils, and faba beans [54]. This process is otherwise termed hybrid extraction [52]. Dry milling involves reducing the size of the product (milling) and then by air-classification [55]. Different milling technologies, for instance, pin [56], hammer [57], roller, stone [58], disc [59], impact, and jet [60], have been reported. The dry-milling technique produces more starch fragmentation and, as a result, a higher percentage of damaged starch, which directly affects starch’s physicochemical qualities [61]. The purity level of air-classified starch can only achieve 65% to 80%, with about 8% to 20% of protein considered as contaminants [54]. Wet milling is accomplished by steeping the grain in water to soften the grain before extraction. The grain is soaked or steeped in water and dilutes sulfurous acid for 24 to 48 h [30], but the process is time-consuming and has a high set-up cost [62]. However, the addition of a reducing agent (*L*-cysteine) has solved this negative effect. *L*-cysteine is an amino acid that is both safe and natural. It is commonly used in food processing as a food additive and acts as a reducing agent [63]. The wet milling fractionation of starch is mostly carried out at a temperature of 40 °C and an autoclaving time of 24–48 h. Different wet-milling procedures (e.g., acid solution, alkaline solution, alcohol) have been reported in several pieces of literature (Table 1). In the alkali method, starch is steeped at a lower concentration, for a short period, and at a lower temperature. While alkali procedures help liberate higher quality starch yields [64] with excellent swelling power, solubility, and water absorption [65], the processing conditions may encourage the growth of putrefactive bacteria [65], which could be suppressed using lactic acid fermentation [66,67]. Although the alkali approach works effectively when it comes to extracting protein from starch, high shear homogenization or water extraction could still be used instead of alkali extraction. Ren and colleagues advocated for future research in order to understand the effect of the extraction agents on the sensory evaluation and functional properties of the extracted starch [52].

Having conducted a critical overview of these distinct wet milling starch extraction types, novel extraction strategies that match the given parameters while also mitigating associated drawbacks are required. Supercritical carbon dioxide (ScCO_2_) and ethanol were used in a study to extract starch from pulses (peas) [90]. The organoleptic qualities of the peas were improved using this extraction process. However, it was suggested that the extraction process’ temperature and pressure be adequately monitored to avoid starch degradation. Liu’s co-workers researched an alternative approach to extracting corn starch using ultrasound-assisted wet milling [91]. Their study revealed that ultrasound-assisted wet milling increased the starch yield by 10% with the same input parameters as traditional wet milling. Similarly, the use of green extraction (GE) captures biodiversity using all renewable energy sources. Green extraction techniques include wave hydro-distillation (WHD), microwave hydro-diffusion and gravity (MHG), ultrasound-assisted extraction (UAE), and microwave-assisted extraction (MAE). At present, we opined that a plethora of ongoing research is currently being carried out to use this technology for the extraction of starch. Regardless of the extraction process chosen, the most important thing to remember is that the recovered starch must be suited to a specific practical application. In summary, we have examined the numerous ways of starch extraction in this section (Section 3). We believe that substantial study on this topic has been documented. Nevertheless, the information presented in this research will also be a helpful tool for researchers in determining the appropriate methods and suitable conditions for starch extraction. Thus, the focus of this review is justified.

## 4. Modification of Starch

Starch modification refers to efforts to increase starch synthesis while also modifying the content and structure of the starch, as well as inducing essential qualities to satisfy specific end goals [32]. According to market study data, modified starch is predicted to reach USD 10,700 million by 2023, growing at a CAGR of 4.2% between 2017 and 2023. In 2016, around 8000 tons of modified starches and other starch components were produced, representing a nearly 50% increase over tons produced in 2015 [92]. These numbers suggest a growing demand for new functional foods and better chances for their development. Because starches have intrinsic flaws, it is necessary to modify them. Physical, chemical, enzymatic, and genetic modifications can all be used to alter starches. Heat (thermal) and pressure (extrusion) treatments, acid hydrolysis, alkali treatment, oxidation, and other methods are commonly utilized for the physical and chemical modification of starch. Research and technological advances have also suggested the use of ScCO_2_ drying as a means of modification of starch.

### 4.1. Physical Modification

Changes in non-digestible carbohydrates could result from physical and chemical starches, affecting their technical qualities and nutritional value. Zieba and colleagues studied an extrusion method for starch modification. The scholars observed a deeper color, more excellent resistance to amylolytic enzyme action, and lower solubility and phase transition heat. They proposed their method as a panacea for overcoming barriers of resistant starch. The physical, thermal, paste attributes, and resistant starch content (RS) of rice and potato starches were investigated after extrusion at 100 °C and 15 rpm with 5% and 10% oleic acid (OA) in the experiment of Cabrera-Ramirez and his co-workers [93]. Findings from their study revealed the presence of granules in a gelatinized starch matrix of the extruded potato starch with 10% OA. Further observation showed that, even after extrusion, the X-ray demonstrated that the structures of rice (orthorhombic) and potato (hexagonal) remained unaltered. The works of Corsato Alvarenga et al. and Zarski et al., among many other researchers, also provided insight into the physical starch modification approach [94,95]. Heat-moisture treatment (HMT), HMT combined with pullulanase (HMT + P), HMT combined with microwave (HMT + M), and HMT combined with citric acids (HMT + A) were used to improve the digestion of whole quinoa flour (WQ) [64,96]. Results showed that all the effects were induced by changes in starch structure, as indicated by the confocal laser scanning microscopy (CLSM) observation of protein and starch together, the decrease in relative crystallinity, and the transformation of starch crystals. Van Rooyen and colleagues [97] have extensively reviewed the different types of heat treatments that disrupt the amylose and amylopectin of starch.

### 4.2. Chemical Modification

Chemical modification is the process of changing the physicochemical properties of starch by introducing new chemical or functional groups into the molecule without changing its structure or size. Various chemical modification methods include oxidation, etherification, esterification, and cationization by introducing some cationic molecules, cross-linking, and grafting among the plethora of chemical modification routes [45]. Regardless of the modification routes, the structure and properties of starch are uniquely affected. For instance, depolymerization of starch occurs as a result of the presence of oxidizing chemicals, resulting in a delay in recrystallization due to the insertion of carbonyl and carboxyl groups [45], whereas the incorporation of hydroxyethyl (etherification) into starch alters the granular structure, enhancing the drug binding potential for various anticancer and other medicines [98]. Meanwhile, chemical processes (e.g., acid hydrolysis) have associated drawbacks to their use. The random attack at the branch point, the high glucose yield, and the difficulty of eliminating the excess acid are disadvantages of the acid hydrolysis of starch [99]. In addition, due to the presence of short-chain molecules formed by acid hydrolysis, Hung et al. found that starch citrate has a decreased water capacity [100]. A double chemical modification (acid hydrolysis and phosphating) of rice starch showed that phosphate starches had better functional and physicochemical properties [101]. Similarly, enzymes such as amylases and proteases have a wide range of uses in starch modification, but maltogenic amylase, which hydrolyzes the amylose portion of the starch, is particularly useful.

### 4.3. Genetic Engineering

In a closed-loop system for starch, genetic modification, seed creation, growth, and delivery to the processing mill are all perfectly controlled. Genome editing [102] and genetic and environmental variation [103] have been reported on the genetic modification of starch. According to our knowledge, there are just a few recently published pieces of literature on the experimental genetic modification of starch. However, crops such as wheat (multiparent advanced generation intercross-MAGIC) [104], rice [105], potato [106], and maize [107], have existing literature reviewed on them. Meanwhile, it is opined that the genetic modification of starch attracts a high regulatory cost [108,109], and, as a result, it is prudent to accept a non-GM starch option. Regardless of the methods used in starch modification, they are supposed to provide the framework needed to address product performance while maintaining structural stability. This will enhance their use for different applications. Some selected starch modification methodologies are presented in Table 2.

## 5. Natural Precursors for Biopolymers

Polysaccharides continue to be most researched, naturally available, and abundant precursors for developing biopolymers [123]. They are composed of several monosaccharides linked together by glycosidic linkages. Some polysaccharides, such as peptidoglycan and cellulose, are found in the cell walls of bacteria and plants, while their counterparts, such as starch and glycogen, are dominant in plants and animals as carbohydrate storage [124]. Of all the polysaccharide components, cellulose and starch are well-rooted in many applications because of their similar chemical structures. Starch and cellulose are both made up of the same D-glucose unit, known as homoglucan or glucopyranose, but they are connected by distinct glycosidic linkages [125]. As narrated in the previous sections, amylose and amylopectin are two forms of biomacromolecules found in starch that are distinguished by their chains. Cellulose is a linear polysaccharide whose chains are highly dependent on the type and treatment of the raw materials (e.g., wood) [126]. The common sources of cellulose materials are bacterial cellulose (BC) [127], microcrystalline cellulose (MCC) [128], and cellulose nanocrystals (CNC) [129]. The complete hydrolysis of MCC shows a high specific surface area. Therefore, MCC is suggested to have high potential for developing a biomaterial [125]. In the field of bio-medicine, cellulose has been employed as a raw material in the making of scaffolds for bone regeneration, artificial blood arteries, temporary skin substitutes, hemodialysis membranes, and controlled medicine release systems [130,131]. In addition, they are widely used for industrial fermentation and bio-energy processes due to their non-toxicity and eco-friendliness. As a result, products such as fiber and packaging materials, thermo-reversible hydrogels, and optical transparent films have been developed using cellulose. On the other hand, starch-based polymers have been profoundly used for orthopedic implants [132], bone replacements, and controlled medication delivery [133]. Starch is a promising biomaterial in food packaging, cosmetics, and diverse composite materials. Despite the promising nature of these two widely used precursors (starch and cellulose) of polysaccharide origin, their applicability is limited. While cellulose has a higher mechanical strength than starch [134,135], their solubility is extremely low [136,137]. Using starch alone is also a major concern because of its inferior properties (weakness). Therefore, some reports have emphasized the development of biobased blended materials. The incorporation of two biopolymers for material creation is an intriguing issue, but recent reports are emerging on their biocompatibility and the characteristics of the resulting materials. Without any dispute of being the most abundant organic polymer on earth, cellulose has gained a lot of attention, closely trailed by starch. A survey of research carried out showed that China, the USA, and France are the leading countries in the world with research outputs on polysaccharide-based biomaterials (e.g., aerogels). The prominence of aerogels is still very limited in Africa, but only Morocco and Nigeria shared a very insignificant number among their counterparts in the world [138]. A plethora of reports, however, exist on the use of starch as a biopolymer, but the possible biomaterials that could be developed using starch (especially those fractioned from pulses) as a precursor have not been widely reported.

## 6. Recent Trends of Starch as Biopolymer

The use of starch for various applications cannot be overemphasized. Starch is the second most abundant polysaccharide on the planet [139] and remains an excellent material in the manufacture of bio-nanocomposites [140,141], such as smart pH sensitive films [142,143] and carbon dots or fluorescent films [144]. Thus, several countries in the world have been expanding their research based on how polysaccharide materials, particularly those that are starch-based, could be integrated into many aspects of human living.

In the previous sections of this review, we have shown how the extraction and modification of starch could be performed. However, the use of these low-value products into biobased products with economic value is of particular interest. Although starch is a prominent and sustainable source for producing biodegradable food packaging materials [145,146], starch-based films have poor mechanical qualities and high stiffness, limiting their applicability in material engineering [147,148]. Blending starch with other biopolymers such as chitosan [149], gelatin [150], polyvinyl alcohol [149], amongst many others, the strength-supporting framework has been investigated to compensate for the starch-based films’ weak mechanical characteristics. Combining two or more polymers yields novel materials with improved mechanical and gas barrier qualities, a viable replacement for synthetic food packaging materials [151]. Starch has traditionally been used as a food ingredient, but it is also being employed in a variety of other uses, including in the manufacture of aerogels and biofoams [19], paper, medicines, and textiles [152]. Nanofillers, film materials, aerogels, and biofoams are biobased materials that provide environmentally friendly solutions. The following section presents information on these starch-based materials.

### 6.1. Nanofillers

Organic nanofillers have been widely used in starch, and the establishment of a hydrogen bonding network has resulted in high interfacial adhesion between the matrix and the nanofiller. Typically, nanochitin, nanochitosan, and nanocellulose have been used as fillers in various applications. Majorly sourced from skeletal components of insects and broadly crustaceans, chitin and chitosan are naturally abundant green materials, and their application with starch-based sources have been documented in several articles [13,153]. The literatures available suggests various processing techniques such as acid hydrolysis [154,155] and ultrasonication [138,156]. Other methods such as mechanical treatment [155], ammonium persulfate (APS) oxidation [157], and 2,2,6,6-tetramethylpiperidin-1-yloxyl (TEMPO)-mediated oxidation [158] have also been proposed for the extraction of chitin.

**Figure 2:**
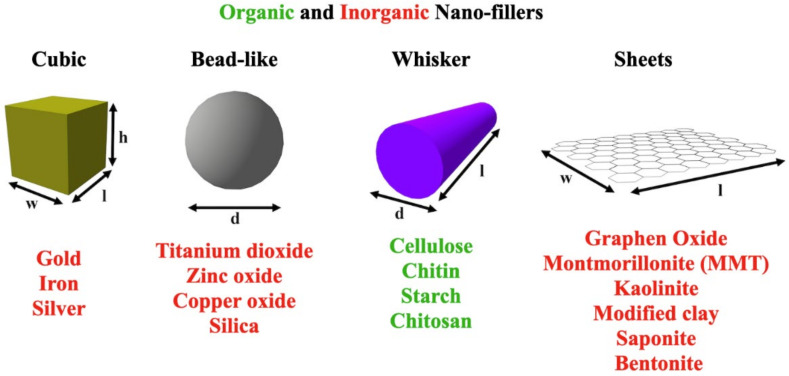
Source of nanofillers as biopolymers (l—length; w—width; d—diameter; h—height).

Outstandingly, the characteristics of starch-based bionanocomposite films are significantly influenced by the shape and size of the chitin nanoparticles. The inclusion of nanowhiskers (nanochitin in the form of nanocrystals and nanofibers) improved the mechanical, thermal, barrier, and antifungal properties of the resulting films. High pressure homogenization was used to extract the additional chitin nanofibers, while acid hydrolysis was used to extract the chitin nanocrystals. Using different weight of starch, and different weights (0, 0.5, 1.0, 2.0, and 5.0%) of nanowhiskers, [159] reported that bionanocomposite films exhibited strong antibacterial activity against Gram-positive L. monocytogenes and Gram-negative *E. coli*. Compared to chitin, chitosan on the other hand is also considered a green material obtained from the deacetylation of chitin and has a higher solubility when dissolved in solvents such as lactic acid, acetic acid, water, acetone, and methanol. Considering the preference for mechanical and chemical stabilities and surface area, nanochitosan presents more advantages, and, because of this, they are adopted as a packaging material in food applications. It is worth noting that the preparation method of nanochitosan is completely different from its counterparts (nanochitin). As reported by Mary and co-workers, the crosslinking of tripolyphosphate and protonated chitosan yielded chitosan nanoparticles, which were employed as reinforcement in potato starch TPS films. The tensile strength of thermoplastic films increased (2.84–10.80 MPa) with the increase of chitosan nanoparticles (0–6%) [13]. Similarly, Dang [160] maintained that, besides the improved extrusion processability characteristics of chitosan, the addition of 0.37–1.45% chitosan to TPS film became stronger and stiffer but less elongated. As established, incorporating nanofillers into starch enhances the mechanical performance of starch-based polymers. Nevertheless, inorganic nanofillers such as nanoclay [161] and carbon nanotubes [162], amongst many others, also have excellent compatibility and good matrix structure, in addition to starch-based materials. In recent decades, material engineers and researchers in the field of nanocomposite materials have been drawn to nanoparticles in spheroidal, platelet-like, and tubular shapes as useful nanofillers [163]. Besides their geometry, their aspect ratio also influences their applicability. This report has been supported by the outstanding findings of some researchers. The sample failure strains were higher in the PI-PM films filled with 10% silicate nanotubes (SNT) and zirconium dioxide (ZrO_2_), suggesting that SNT and ZrO_2_ may be more successful in enhancing the ductility of the polyimide nanocomposites for applications where the more brittle polyimide/MMT nanocomposites films are unusable. In addition, in oriented nanocomposite fibers, Ivankova and colleagues reported that vapor-grown carbon nanofibers (VGCFs) and single-wall carbon nanotubes (SWCNTs) boosted tensile strength and modulus by 275 MPa and 5 GPa, respectively, but decreased deformation at break. The researchers discovered that SWCNTs are more effective reinforcers than VGCFs [164]. More scholarly reports have addressed the role of the geometry of nanocomposite and filler materials and are limited to interesting topics related to their toughness, strength, and stiffness characteristics [165,166] and their dielectric properties [167]. Figure 2 shows the geometry and aspect ratio of common nanofillers.

### 6.2. Intelligent Films

Consumers can benefit from better preservation of food materials due to smart packaging using starch-based materials, thus serving as an excellent response to mitigate the preservation of foods using chemicals. Starch, particularly corn starch, is considered one of the most promising biopolymer materials since it is abundant, inexpensive, biodegradable, tasty, and has good film-forming potential [168,169]. The mechanical properties of starch-based films can be considerably improved by combining them with other biodegradable polymers, such as polyvinyl alcohol (PVA), that are better suited for film and coating production. Novel applications, such as intelligent pH-sensitive packaging, are attracting interest from scholars. Though relatively known at present, Ezati and co-workers posited that intelligent pH-sensitive films could serve as a cutting-edge technology whereby food samples could easily be measured in real time for freshness without delay [143]. Intelligent packaging films may be natural, such as alizarin dye [143], anthocyanin [170,171], and curcumin [172], or synthetic, but the former is widely used in assessing the color change of foods. The most commonly used natural intelligent packaging film is anthocyanin, which is dominant in agricultural materials such as butterfly pea flower [171], black plum peel [173], and purple potato [174], amongst many other sources. Several authors have reported outstanding results using starch-based sources as intelligent packing films.

To mention a few, the addition of anthocyanin-rich bayberry extract to cassava starch produced an excellent colorimetric film with exceptional antioxidant properties and tensile strength [175], whereas incorporating *L. ruthenicumanthocyanins* into cassava starch enhanced the water vapor permeability and antioxidant potential and reduced the ultraviolet light barrier of the starch film [176]. In a more recent study, the researchers developed and compared starch-based films with various anthocyanin extracts. The color response of the purple sweet potato (PSP) and red cabbage (RC) extracts films were distinct in monitoring the deterioration of shrimp. The color response and mechanical qualities of PSP film were superior to those of RC film [169].

In order to critically assess the mechanical properties of pH-sensitive films, Mei and colleagues evidenced that the flexibility of sago starch increased with the addition of anthocyanin obtained from torch ginger extract (TGE) [177]. In their study, tensile strength (4.26 N/m^2^), toughness (2.54 MJ/m^3^), Young’s Modulus (73.96 MPa), and water vapor permeability (0.00026 gm/day. kPa.m^2^) were all lowest in the film with the highest concentration of TGE. However, when compared to other films, it had the highest elongation at break (85.14%), moisture content (0.27%), and water solubility (37.92%). The films containing TGE extract change color when the pH is changed, according to pH sensitivity analysis. As the pH increased from 4 to 9, the color of the films changed from pink to slightly green [177]. Properties such as water contact angle, moisture content and surface moisture, and total surface energy of intelligent pH-sensitive films have rarely been studied [178]. However, a recent study revealed that intelligent, sensitive films also have a magnetic field and enzyme responsive characteristics [179].

### 6.3. Aerogels

**Figure 3:**
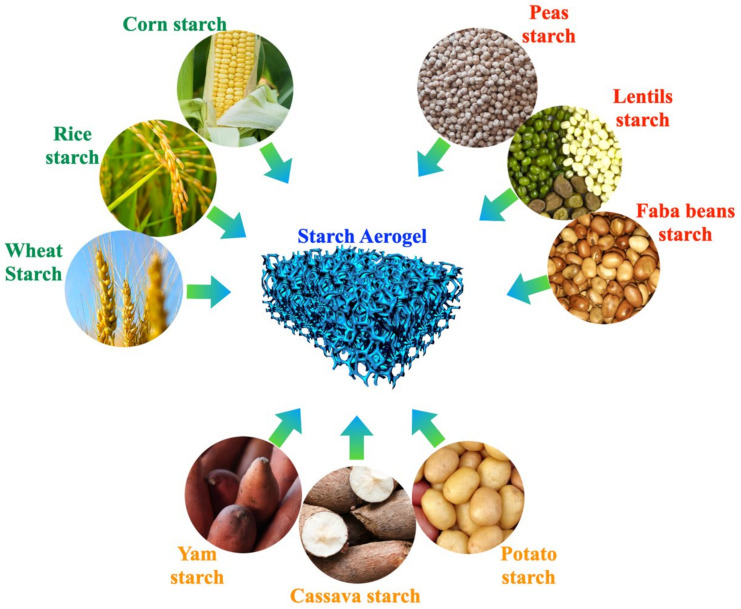
Potential starch sources for aerogels.

Aerogel is a solid, ultra-lightweight substance with high porosity and other exceptional features [180]. It consists of a three-dimensional and very porous network created from organic, inorganic, or mixed precursors utilizing a sol-gel approach paired with quick-drying technology to extract the liquid in an alcogel and replace it with another liquid [181,182]. However, in a more generic term, any non-fluid colloidal network or polymer network that is enlarged across its entire volume by a fluid could be referred to as aerogels. The study revealed that gels have a variety of properties that are not solely dependent on the drying procedure. Kistler was the first scientist to create aerogels using silica, stannic oxide, and cellulose in 1931. The procedure involved replacing the liquid (solvent) in a hydrogel with air without changing the network structure in the hydrogel state. The sol-gel process was used to create the gel, and the solvent was removed by drying it at supercritical temperatures. Capillary pressure, which happens during drying and is responsible for pore collapse, is theoretically zero in the supercritical state because no liquid-vapor contact (no meniscus) arises. The experiment produced a low density, high specific surface area, low thermal conductivity, and other unique properties such as shock absorption. Many scholars are interested in the biodegradability and biocompatibility of aerogels because conversion processes on natural biodegradable materials could address environmental concerns. Kistler’s research triggered the interest of many scholars who were particularly interested in the biodegradability and biocompatibility of aerogels. Since then, cellulose has been used to replace silica or synthetic polymers as a structural material, and it has been proven to provide mechanical support to biobased products [183,184]. Proteins, starch, sugar cane, alginate, chitosan, and vegetable oils have been used as biobased components for aerogels [185]. Figure 3 shows the potential starch sources for the fabrication of nanocomposites such as aerogels.

Aerogels are distinguished by their drying process and precursors. The drying techniques necessitate the use of specific parameters and precursors in the production of aerogels. Broadly, organic and inorganic aerogels are the two types of supercritically-dried aerogels. However, recent research combined organic and inorganic aerogel precursors, resulting in hybrid aerogels resulting in different aerogel nomenclature such as ceramic, carbon, cellulose, and clay. Given these differences, it is necessary to distinguish existing aerogels and their possibilities in industrial application.

#### 6.3.1. Silica/Ceramic Aerogels

These sorts of aerogels were the forerunners of today’s aerogels. They were first described by S.S. Kistler, who used the supercritical drying (ScCO_2_) process in the sol-gel processes of silicon and vanadium oxide (V_2_O_5_). Tetramethoxysilane (TMOS) and tetraethyl orthosilicate (TEOS) are typical precursors in silica aerogels. TMOS is dissolved in methanol, then hydrolyzed and condensed in a precise amount of water in this method. Recent studies have reported the use of silica and metal oxide aerogels. Silica is an exciting material for high-performance insulation applications due to its aesthetics, non-flammability, non-reactivity, and low thermal conductivity [63]. However, they have two fundamental flaws: weak mechanical properties, high susceptibility to cracks, and worries regarding long-term economic viability [186]. The precursors for metal oxide are chosen based on their architectures (V_2_O_5_—fibrous and Al_2_O_3_—leaf-like). At comparable densities, metal oxides such as ZrO_2_ have a higher Young’s modulus (10.7 MPa), whereas most common SiO_2_ materials have a modulus of 2 to 3 MPa.

Schafer’s colleagues synthesized inorganic aerogels via rapid gelation using chloride precursors. Their methodology was assumed a new facile approach because of some outstanding observations drawn in from their experiments. As revealed by nitrogen adsorption experiments, supercritical drying of the wet alcogel in CO_2_ produced aerogels with BET surfaces up to 1390 m^2^/g and hydrolysis reaction and gel formation can be regulated by adding crosslinking agents such as propylene oxide, diluting with alcohols, or beginning from a water glass. The water glass serves as a low-cost network maker and provides precise pH control by neutralizing acid-forming hydrolysis processes [187]. Carbon fibers have been used to improve the stability of ceramic aerogels [188]. Carbon-based nanomaterials (CBNs) such as active carbon (black carbon), carbon nanotubes, carbon nanofibers, and graphene have piqued researchers’ and the industry’s attention.

The application of these enhanced carbon nanomaterials in the FD process to make aerogels has lately been widely documented. Micropores of carbon nanofibers and carbon nanotubes had dimensions ranging from 1.8 to 2.0 nm, whereas mesopores and macropores had diameters ranging from 2 to 250 nm. In addition, the specific surface area ranges from 164–186 m^2^/g [189]. Parameters such as skeletal density, refractive index, and the thermal conductivity of silica aerogels could be determined in silica aerogels. Based on our standpoint, there has been limited interest on the sound velocity measurement of aerogels.

#### 6.3.2. Clay Aerogels

Aerogel of this form was created using low-cost, non-toxic components using an environmentally favorable freeze-drying technology, making them more desirable for sustainability and environmental issues. Clay aerogels are distinguished by their low density (0.1 g/cm^3^), high porosity, and low thermal conductivity (0.020.05 W/m K) [190], but they are limited in their ability to provide excellent mechanical support; therefore, reinforcing becomes a panacea for their applications. The growing interest in clay aerogels has resulted in several good publications on the crucial factors (e.g., freezing rate and clay concentration) affecting clay aerogels. The microstructure, wettability, moisture resistance, mechanical properties, and thermal conductivity all affect the mechanical performance of clay aerogels [191]. Clay aerogel moisture absorption was lowered by up to 40% in their study while maintaining excellent dimensional stability. The aerogel composites feature a contact angle of 140°, a 93% reduction in water absorption, a compressive modulus of 3.2 MPa, and a low thermal conductivity of 0.038 W/m^2^ (m K). In the freeze-drying process, the compressive strength and fracture resistance of CNF/starch/clay aerogels were enhanced at reduced moisture (2%) without compromising the structure of the aerogel. The density (0.05 g/cm^3^) and thermal conductivity (41.5 mW/mK) were drastically lowered [192]. However, it is worth noting that attempting to overcome a constraint of inorganic aerogels such as clay aerogels invariably results in a loophole that necessitates practical solutions. Several studies that were carried out to address these restrictions led to the formation of polymer-based (organic) aerogels. This points to the possibility of new advances in the use of aerogels in a broader range of applications.

#### 6.3.3. Polymeric Hybrid Aerogels

The demand for polymer aerogels arose from industries’ unquenchable quest for materials with extremely low density, outstanding thermal characteristics, and a large specific surface area (aerospace). National Aeronautics and Space Administration (NASA), for example, has a high demand for materials such as polyimides due to their insulating qualities [193]. Polymer (polyimide) aerogels are highly flexible and moisture resistant and can be shaped into valuable films and fabrics. Precursors such as 2,2-dimethylbenzidine (DMBZ), 1,3,5-triaminophenoxybenzene, p-phenylenediamine (PPDA), 4,4-oxydianiline, and biphenyl dianhydride (BPDA) are widely accepted in the manufacturing of polymer aerogels [194]. Polymeric aerogels can be modified in two ways: by adding polymers or by introducing renewable feedstocks (biobased sources). Aerogels produced from both routes mostly employ the ScCO_2_ drying process. Nonetheless, freeze-drying is also applicable to polymer aerogels. Uniquely, supercritical drying presents higher porosity, while the freeze-dried technique, on the other hand, is frequently used because of its environmentally benign qualities and inexpensive cost. Extant studies on the supercritical drying of aerogels use biobased materials such as alginate, chitin, chitosan, carrageenan, agar, and cellulose gels as part of their component [28]. Nevertheless, information about their inherent characteristics for industrial capacities is relatively unknown. The morphology of the resulting ScCO_2_-dried aerogels from these biobased sources was determined to be macroporosity (pore width >50 nm), mesoporosity (2–50 nm), and microporosity (less than 2 nm) during the examination of the synthesis and characterization of tannin/lignin-based aerogels [195]. Class I hybrids are created by mixing two independent organic and inorganic components in a specific sol-gel solution, whereas class II hybrids are created using organ replacement inorganic precursors or silylated polymers. Class II aerogels have shown higher physicochemical stability and controllability in microstructures and physical properties than class I hybrids, which is a distinct difference between the two classes of polymer-induced hybrid aerogels [196].

### 6.4. Effect of Crosslinkers

Starch could be obtained from common agricultural materials such as rice, wheat, corn, potato, cassava, etc. [197,198]. Though underutilized, recent research has made a significant contribution to the use of starch. In the polymer aspect, starch fits well as a biobased material for aerogels, and a plethora of studies showed their effectiveness. In all the surveys of literature, corn and potato starches are more prominent for aerogel productions, and there has been slight preference for other starch, particularly those obtained from pulses. To improve the gel structure of starch, crosslinking agents such as glutaraldehyde and trisodium citrate have been used, but the former have peculiar limitations such as high toxicity, which may limit their applications, especially in food applications.

Trisodium citrate prepared by the addition of citric acid to an alkali solution has a better reaction when incorporated into the gelatinization process of aerogels because of their eco-friendliness and non-toxicity. Using a freeze-drying method, Camani and co-workers prepared starch-based aerogels using corn starch and trisodium citrate as the crosslinking agent at different levels of concentration. The authors evidenced a microporous structure (>50 nm) but observed that a higher concentration of crosslinking agents led to a larger pore diameter, which invariably affects the porous structure [199].

**Figure 4:**
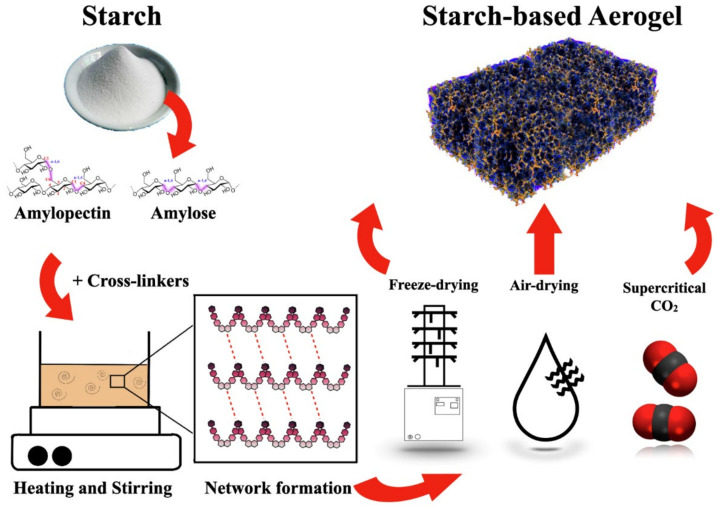
Illustration of crosslinkers in aerogels.

The amylose content of starches (potato and peas) was varied for starch-based aerogels [200]. The authors evidenced high specific surface area, low density, and a thermal conductivity range of 0.021–0.023 W/m K for pea starch but emphasized the importance of retrogradation time, which decreases the specific surface area and improves the thermal conductivity and other mechanical properties. Figure 4 shows a schematic representation of a crosslinking process for a starch-based aerogel. Besides, the pore structure of aerogels also makes them uniquely fit for various applications. Aerogels require a particular amount of open pore structure to inhale pollutants and a certain amount of closed pore structure to store pollutants as an adsorbent substance [138]. Therefore, as a result, the importance of aerogel structure design is to better adapt to various application needs.

## 7. The Manufacture of Aerogels

As discussed, aerogels could be organic, inorganic, or hybrid. The transition between these classes of aerogels is triggered by possible limitations associated with the inorganic aerogel, which S.S. Kistler first synthesized. The development of organic aerogels also has drawbacks related to their weak mechanical framework. Thus, to address these overall limitations, hybrid aerogels became a panacea. Though it could be assumed that the advent of hybrid aerogels has met the setbacks of organic and inorganic aerogels, the mechanical properties of polymeric aerogels must still be methodically tailored. Some scholars have undertaken efforts to manipulate the density and pore volume of polymeric aerogels, primarily by altering the precursor polymer concentration [201]. Therefore, this section of the review paper aims to provide information about the key parameters and processes that influence aerogel production. In doing this, gelatinization, drying operations, and mechanical properties of these aerogels will be discussed.

### 7.1. Solvent Gelatinization

The manufacturing of aerogels via the sol-gel method has quickly become a fascinating new subject of material science research. Practically all aerogels are made using the sol-gel route [180,202]. This process essentially involves four key stages: solvent preparation (creation of an aqueous suspension through the dispersion of solid conglomerates in a liquid), gelation (crosslinking and branching), aging (mechanical process to increase strength), and drying (careful removal of the solvent). The precursor is dissolved in a solvent (water or alcohol) with an acidic or basic catalyst and then converted into a colloidal gel structure by a series of hydrolysis and polycondensation reactions. Solubility of the precursor offers a great advantage in the gel formation, while physical interactions such as van der Waals forces, hydrogen bonds, hydrophobic or electronic associations, and chain entanglements cross-link physical gels [180]. The concentration of the solution or dispersion and the temperature determines the gelation speed of physical gels using cellulose as a precursor. Chemical cross-linkers, which may create covalent bonds between polymer chains during gelation, frequently provide cellulose gels with a stable structure and effective swelling [203].

In applying the sol-gel process first used for the production of silica aerogels, the excellent hydrophobicity and flexibility of aerogels (e.g., silica) are critical for long-term usage. We discovered that the sol-gel technique is highly prominent and the only method reported so far in forming a three-dimensional (3D) porous network structure in an aerogel after reviewing published articles on the subject. However, sol-gel techniques have several drawbacks, including high material costs, extended processing times, and poor mechanical properties. Nonetheless, due to its flexibility, which allows for the adjustment of the pore structure, it is one of the greatest approaches for generating extremely porous solids.

### 7.2. Aerogel Drying

Removing the underlying liquid in aerogels without disturbing the solid structural framework is vital in aerogel synthesis. The purpose of drying solid materials is to remove moisture from their pores, which would otherwise cause the silica framework to cave in and succumb, causing the gel structure to collapse completely [204]. Among the numerous drying methods, such as supercritical drying, ambient-pressure drying, freeze-drying, vacuum drying, and microwave drying, as reported in the literature [181], only three drying methods (freeze-drying, ambient air (pressure) drying, and supercritical carbon dioxide) have been used for the drying of gel-like materials [205]. Nevertheless, freeze-drying and supercritical carbon dioxide are preferred because they do not harm the underlying microstructure of aerogels [181]. Meanwhile, evaporation could also be regarded as a drying process for gels [204]. All gel-like materials are referred to as aerogels in this review, irrespective of their drying process. However, the thrust of this section below reveals peculiar characteristics of these drying methods.

#### 7.2.1. Freeze-Drying (FD)

Freeze-drying or lyophilization releases solid, generally frozen, water from the pores of a wet precursor [206]. Freeze-drying involves two notable processes: freezing and two stages of drying. In freezing, the prepared gel is frozen at a temperature below the freezing point (usually between −50 and −85 °C) of the liquid medium (typically water) [206]. After this process, the liquid is evaporated mainly by sublimation, thus preventing structural collapse and shrinking. Sublimation is removing the solvent from its vapor state by lowering the pressure. To achieve solvent sublimation and low-pressure levels, a higher temperature and lower pressure are required at this stage. The factors influencing the sublimation rate in aerogel production include the concentration of the precursor, gel size and form, and temperature. The two stages of dryings are primary (initial) and secondary (final) drying [207]. The initial drying removes a more significant proportion (about 95%) of all the water present; simultaneously, the pressure and temperature are controlled differently in the final drying than in the initial drying. While the pressure drops, the temperature rises to eliminate any unfrozen or bound water deposits in the material [17]. In most circumstances, the ultimate goal of the final drying is to achieve a water content of 1 to 4% [208]. However, the sublimation rate during FD is generally observed to be prolonged. Several authors have reported the effect of freeze-drying porous solids (organic, inorganic, hybrid) with foresight into their applications. Hot starch material melted frozen at 20 °C had a maximum specific surface area attained by the freeze-drying of 7.7 m^2^/g [209]; while using CNFs/Na_2_SiO_3_ as precursors in the freeze-drying process, an ultralight aerogel with a pore volume capable of absorbing about 1.49 mmol/g of CO_2_ was produced [210].

#### 7.2.2. Supercritical Carbon Dioxide (ScCO_2_) Drying

The most important step in the production of aerogels is supercritical drying, which preserves the three-dimensional pore structure, resulting in unique material properties such as high porosity, low density, and large surface area [211]. This process, however, is dictated by the selection of drying solvents. Organic (e.g., acetone, ethanol, methanol) and inorganic (e.g., water, CO_2_) solvents have been used in most scenarios [212]. In supercritical drying, water, for example, serves as a strong mineralizer for inorganic materials, primarily amorphous silica, under supercritical conditions. On the other hand, water has limited use in supercritical drying, but it is widely used as a gel-initiating solvent. In addition, alcohols, ether, methanol, and acetone are used because of their liquid-like densities. Meanwhile, ether and acetone are rarely employed due to safety concerns; in contrast, the toxicity of methanol limits their applications, especially when aerogels are considered for industrial applications such as food and pharmaceuticals. In ScCO_2_ drying, thermodynamic parameters such as pressure and temperature can alter the gel’s liquid phase condition. As a result, ScCO_2_ drying has been discovered to be the ideal drying procedure for replacing the liquid with gas in the pores of the produced gels solely because CO_2_ is non-reactive and has a critical temperature that is very close to room temperature. In addition, CO_2_ benefits from its mild supercritical conditions, particularly the 304.13 K supercritical temperature. Sahin’s co-workers reported the ScCO_2_ procedure and stressed the importance of the solvent requirement and its benefits [211]. As a result, water is replaced with a solvent with a high solubility of ScCO_2_. Solvent exchange can cause gel shrinkage due to solvent-gel interactions. The type of solvent used to reduce shrinkage is very important. The critical parameters of an organic solvent are temperature (K), pressure (MPa), density (kg/m^3^), molar volume (cm^3^), compressibility factor, and permittivity [213].

Aerogels manufactured by ScCO_2_ drying have a strong affinity for moisture over time, causing a capillary effect and eventually resulting in textural degradation. As a result, some researchers who have modified the supercritical drying process arguably emphasized that ScCO_2_ is inefficient, hazardous, costly, and unscalable. Nevertheless, ScCO_2_ drying has thus been the mainstream drying process of gel substances mainly utilized in functional areas such as medicines, culinary science, and the textile industry.

#### 7.2.3. Ambient Pressure Drying (APD)

Although APD is a less energy-intensive alternative, it frequently relies on refilling the initial gel preparation solvent [214]. Cheng described the APD of silica aerogels, but little attention had been paid to the effects of gel treatment, drying temperature, and modifying agent molar ratio in APD aerogels [215]. The main advantage of ambient pressure drying over other aerogel-making drying procedures is that it does not require dangerous high-pressure equipment; however, it requires numerous steps of solvent exchange and chemical reaction [216]. Using APD in the production of aerogel, Kim’s experiment produced an aerogel with a specific surface area of 1028 m^2^/g, a total pore volume of 1.2 cm^3^/g, an average pore diameter of 5.5 nm, a penetration ratio of 70%, and a thermal conductivity of 0.02 W/mK [217]. In a similar study, industrial solid wastes and dislodged sludges via APD were used in the manufacturing of aerogels. Results evidenced a pore volume of 3.3 ± 0.1 cm^3^/g, with a pore size of 18.5 nm, a high hydrophobicity of 144.2 ± 1.1°, and low thermal conductivity of 0.031 ± 0.001 W/m.K. [218].

**Figure 5:**
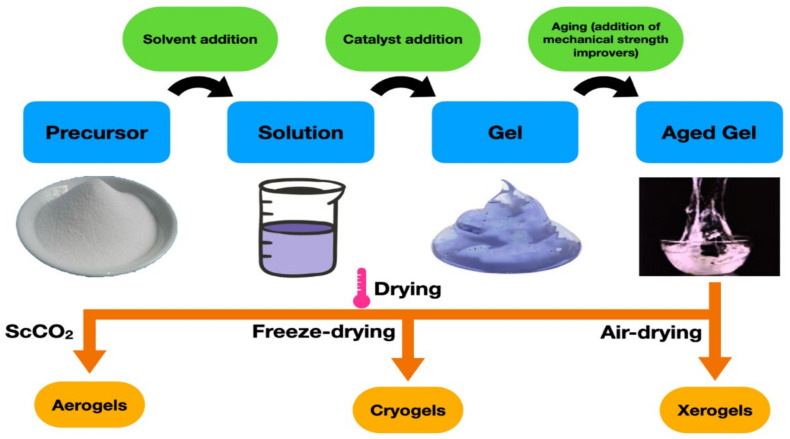
Process flow procedures for aerogel production.

Although water is largely not used in this drying process, except as an initial solvent for the precursor, scholars researched the eco-friendliness of water-based hydrophobicity silica aerogels using the APD route [219]. Specifically, morphology, structure, and hydrophobicity were their points of interest. Outstanding findings revealed that the gelation time of the manufactured aerogels decreased from 40 min to 2 min, and the shrinkage of the aerogel sample decreased from 15.5% to 3.5% when the pH value was increased from 7.5 to 10. With a water contact angle (WCA) of 160.6 ± 1.3°, the produced silica aerogel demonstrated exceptional anti-adhesion capabilities and superhydrophobic ability. Khedkar and colleagues also employed a cost-effective and safe ambient pressure drying approach to make hydrophobic silica aerogels from silicic acid with pH variations. Their result revealed that all of the samples of the aerogels produced were hydrophobic. However, the sample with a pH of 5 had better outcomes in terms of optical transmittance, thermal stability, hydrophobicity, and surface area when compared to the other pH varied samples [220]. Figure 5 illustrates the laboratory process for the manufacturing of aerogels.

In the drying of cellulose aerogels, FD is widely employed, while ambient pressure drying is rarely used. The porosity and specific surface area of freeze-dried material from nanocellulose sources could rise as high as 99.97% and 700.1 m^2^/g, respectively, compared to ScCO_2_ drying with a porosity and specific surface area of 95% and 353 m^2^/g, respectively. The density of FD was as low as 0.003 g/cm^3^, which is much lower than ScCO_2_ (0.08 g/cm^3^) [180]. All developed starch aerogels via ScCO_2_ drying had specific surface areas between 60 to 100 m^2^/g [221,222] but could go as high as 254 m^2^/g [196]. Technically, processing parameters (retrogradation temperature, drying condition, methods, and amylose content) and morphology (porosity, density, and specific surface area) are critical to the manufacturing and production of aerogels [139]. Although the effect of the processing parameters on the outcome of aerogels is attracting attention in academia, there is limited information about the kinetic studies in aerogel making.

## 8. Biofoams and Bioplastics

Non-biodegradable synthetic polymer-based foams, such as polyethylene (PE), polystyrene (PS), polyurethanes (PU), and polypropylene (PP), are known to cause more environmental issues than their biodegradable counterparts [107,223], making biodegradable polymer foams a viable alternative. The viability of producing foams from biomass has been investigated in several studies. The majority of these studies looked at the possibility of manufacturing polyurethane foams from agricultural waste, particularly those that had been extracted and contained starches and oil proteins. When comparing biodegradable packaging to synthetic materials in terms of cost, biodegradable packaging created from bioproducts is not always the most cost-effective option in the short term [224]. As a result, agricultural or industrial by-products may be used to create novel packaging materials. Biofoams are ultralight, low-density biomaterials that are growing rapidly in material applications for sustainable development. In recent investigations and dating back decades, starch-based and polylactic foams have received a lot of interest. Biofoams manufactured from starches and polyesters such as polylactic acids (PLAs) offer immense industrial promise, and starch foams have the potential to replace polystyrene-based food trays [225]. Potato [224], wheat gluten [226], coconut fibers and sugar bagasse [227], canola [228], and rice husks [229] have all been examined for biofoam production. However, because raw starch has limited applications, particularly in areas where strength is critical, the introduction of mechanically improving components encourages the use of starch in a biofoam. As a result, numerous researchers found that mixing starch with various plasticizers (natural or synthetic) and additives, such as natural fibers, could result in new foam-like polymer composites with better properties in their report. Rice starch-based biofoams showed higher density, tensile strain, and maximum flexural stress values [230]. Under various loadings of microcrystalline cellulose from the banana stem, the hydrophobicity and biodegradability of starch-based biofoam reinforced with microcrystalline cellulose from the banana stem were improved [231]. Some scholars have also used melt extrusion with water as a blowing agent in the manufacturing of starch foams. Natural fibers (hemp, cellulose, cotton linter, sugarcane, and coconut) were added to the starch foam, resulting in a density reduction of up to 33%, decreased water absorption, and improved mechanical properties, depending on the fiber concentration and type [232].

Biopolymers (e.g., bioplastics) derived from renewable resources such as plants and animals can assist in addressing both the challenges of diminishing oil reserves and the environmental issues associated with the rising use of petroleum-based plastics [233]. In economic terms, it is expected that the demand for bioplastics will increase by 19% in 2022 compared to 2017 [234]. Though the bioplastic business still has low commercial value, efforts are geared towards ensuring its sustainability in society [234]. A plethora of studies has been published in recent years in an attempt to lower the volume of non-biodegradable polymers, whereas the advent of biodegradable polymers is gradually attracting keen interest. The primary drivers of the rapid increase in bioplastics are biodegradable PBAT (polybutylene adipate terephthalate), PBS (polybutylene succinate), and biobased PAs (polyamides), PE (polyethylene), PLA (polylactic acid), and PP (polypropylene). By 2027, biodegradable bioplastics are expected to account for 69.6% of total bioplastic output and demand, compared to 30.4% for nonbiodegradable bioplastics [233]. PBAT and cellulosic film are expected to be the most (30.4%) and least (1.2%) commercially necessary biobased biodegradable films, respectively. On the other hand, other sources employ a far lower amount of data (0.4%) [233]. In summary, bioplastic research has primarily concentrated on starch-based sources. Although weak mechanical properties threaten starch bioproducts, the absence of a suitable plasticizer also challenges their processability and storability. As a result, natural fibers are a panacea to these limitations. Natural fiber (e.g., cotton, hemp, sisal, jute, flax, coconut, sugarcane, etc.) increases the mechanical strength, stiffness, and heat conductivity of bioplastics.

### 8.1. Thermoplastic Starch

TPS, also known as plasticized starch, is a substance generated by altering the structure of starch granules. It is recognized to have various advantages, including renewability and flexibility in the manufacturing process. The global thermoplastic starch market reached 179.58-kilometric tons in 2019. Between 2020 and 2025, the market is expected to reach a value of 255.82-kilometric tons, with a CAGR of 7.01% [235]. Despite its good global market figures, TPS has been connected to some disadvantages, including poor mechanical qualities and retrogradation. Furthermore, the brittleness of TPS is exacerbated by plasticizer migration into the environment. Starch loses its granular shape and crystalline structure during the TPS manufacturing process. This destructuration is performed by heating and shearing starch grains and adding a plasticizer. In other words, the critical components that aid thermoplastification are starch, plasticizer, and thermomechanical energy. The thermoplastification of starch involves the formation of hydrogen bonds between the plasticizer and the starch in the presence of energy. In most cases, this process disrupts the hydrogen bonds between the hydroxyl groups of the starch molecules, resulting in the collapse of starch crystallinity. A plasticizer is a chemical added to a plastic material to make it more flexible and adaptable. Plasticizers work by causing the hydrogen bonds between starch polymers to break and replace the polymer chains to move freely [236]. They can be classified as internal or external polymers from the polymer viewpoint. External plasticizers are a low-volatile chemical added to polymers that reacts with the original polymer or is crosslinked into the polymeric matrix [237]. Plasticizer molecules penetrate starch granules and destroy the starch’s core hydrogen bonds when exposed to high temperatures, pressures, and shearing. TPS is made with a variety of plasticizers (TPSs) [11].

According to many reported studies, sorbitol, glycols, maltodextrin, amino acid, citric acid, formamide, and urea are the most commonly used plasticizers for TPS [94]. However, water is the most common solvent or plasticizer in starch, while glycerol is also used frequently [238]. Although the breakage of hydrogen bonds in starch promotes crystallinity loss, this occurs only when the water content is considerable (10–30% *w*/*w*). Biodegradable thermoplastic starches such as polybutylene succinate adipate (PBSA), polyhydroxybutyrate (PHB), and polycaprolactone (PCL) provide outstanding flexibility, a low melting point, and good compatibility [239]. Succinctly, most plastic materials aim for industrial applications, including food packaging, coatings and adhesives, agriculture and horticulture, and consumer goods; breaking down their barriers is critical. Such constraints, however, could be overcome by using polymer blends.

### 8.2. Starch-Filled Polymer Blends

Polymer blending is the incorporation of hydrophilic starch with hydrophobic polymers (e.g., polyethylene). Starch-filled biodegradable polymer blends to replace inert plastic materials have attracted considerable interest due to the compatibility and degradation drawbacks of filler starch [240]. Compatibilization, or the melting of two or more polymers, is a rapid technique to provide materials with a broader range of properties while avoiding the limitations of individual components. Nonetheless, there are two major compatibilization routes for starch-filled polymer blends (miscible and immiscible) [240]. Copolymers (ex-situ), reactive graft polymers (in situ), and radical processing are the three main paths for this procedure (dynamic vulcanization). Thermoplastic elastomers, such as styrene-ethylene-butene-styrene block copolymer (SEBS) and ethylene-propylene-diene (EPDM), have been used for compatibilization [241,242]. Reactive copolymers on immiscible polypropylene (PP)–polystyrene (PS) blends with varying PS concentrations using mechanical (tensile and tensile impact), rheological (melt flow rate, extensional and dynamic rheology), and morphological (scanning electron microscopy) analysis (10 wt. %) have been studied [243]. A maleic anhydride-grafted-PLA (GMAPLA) coupling agent and its concentrations (5, 10, and 15%) had an influence on the mechanical and thermal properties of PLA–TPS blends [244]. According to their findings, the addition of GMAPLA improved the mechanical qualities of TPS/PLA blends (tensile, flexural, and impact strength), but blending with 10% GMAPLA demonstrated substantial gains in strength properties among the three concentrations. Akrami and colleagues synthesized the compatibilizer in two steps in another work. The first stage was to melt combined MA and PEG (4:40 *w*/*w* ratio) in a flask at 130 °C for 2 h with agitation. Second, starch particles (56 wt%) were added to the PEG and MA mixture, and the reaction was kept at 150 °C for an additional 2 h. The compatibilizer improved interfacial adhesion, which they discovered was due to interactions between the compatibilizer’s free end carboxylic acid groups and the active groups of the TPS and PLA phases [245]. Since the mechanical qualities of polymer blends are of peculiar interest, previous studies have uniquely emphasized the mechanical properties of polymer blends with respect to their elongation, brittleness, and ductile transition as affected by processing techniques [246]. For instance, the addition of PCL to rubbery TPS increases the modulus but decreases the yield strength [247]. Solution casting, extrusion, injection molding, compression molding, and hot pressing are some of the preparation procedures that have been offered in the manufacturing of polymer blends, and special reports have backed up their relevance. Extrusion is the most common process for starch-based polymer bends, followed by solution casting. These processes are further discussed in the next section of this review paper. A summary of preparatory techniques documented in a few studies is summarized and presented in Table 3.

## 9. Manufacturing Process for Biofoams

This section and subsequent sub-sections highlight the processing methods for the manufacture of biofoams. Mechanical and thermal processes are the two main categories of these processes. However, because the drying processes of aerogels and biofoams are nearly identical, the drying process of aerogels is partially helpful in this regard. As a result, we are more concerned with the mechanical aspects of biofoam production. The preferred methods of producing biofoams include extrusion, injection, and compression molding (baking) [269]. Foaming agents are also used to manufacture biofoams to make the process cleaner. In the sub-section below, we go over them in greater detail.

### 9.1. Extrusion

Extrusion is the process of compressing, conveying, and expelling a material through a specially designed outlet. Extrusion creates high pressure near the die output because of the expansion caused by injected gas or in situ generated gas. The pressure of the material drops significantly as it comes into contact with ambient air, causing the substance to foam. The gas bubbles that form in the starch paste are predominantly carbon dioxide (CO_2_) and nitrogen (N2). However, the type of gases produced are dependent on the utilized blowing agent [270]. Extrusion foaming involves six stages: the plasticized flow of polymer melts in the extruder; dissolution and homogenization of the blowing agent in the polymer melt; cooling optimization process of the polymer/blowing agent solution by lowering the temperature to a suitable foaming temperature; shear and elongation flow of the polymer/gas homogenized fluid inside the die channel; and diffusion-induced growth of bubbles [271]. However, these stages can be broken down into three main steps: nucleation, bubble foaming (stabilization), and foaming growth [272]. The cellular structure of the foam can be significantly influenced by the conditions of the extrusion process, such as speed of the screw, the temperature of the barrel and size of the die, and compositions of material such as moisture in the feed, type of starch, and concentration of nucleating agents as reported in the literature [273].

Typically, the temperature of the barrel is maintained at 120–170 °C and the speed of the screw at 70–400 rpm in starch-based foam extrusion [274]. The number of cells rises with increasing screw speed and barrel temperature, resulting in the higher expansion ratio of the foams [275], but the process leads to the formation of lower density foams [276]. Capezza and colleagues carried out an extrusion process on wheat gluten (WG) foam at a 50 wt. % aqueous WG mixture. Their findings revealed the optimum temperature range of 80–120 °C, yielding extrudates with pore diameters ranging from 65 to 116 m, with both open and closed pores. Furthermore, the authors revealed that the best mechanical qualities were found in wheat starch foams, especially when they were aged. The change in the extrusion processing parameters using the best ratios of wheat starch/glycerol/gluten/sodium bicarbonate (100/46/25/1) resulted in a higher and more stable expansion (9.1) at a screw speed and input rate of 300 rpm and 21 kg/h, respectively. In general, a feed with a water content of 15–18% is regarded best for producing foams with the maximum expansion ratio [277]. Extrusion for biofoam manufacturing will necessitate further experimental and modeling studies to improve theoretical knowledge and technical processes, as it is a necessary operation in practically all industrial applications.

### 9.2. Compression Molding/Baking

Compression molding is one of the techniques used to create a wide range of composite materials. This process involves the use of pressure, which strongly influences the strength and complexity of the starch material. More so, compression molding has high-volume productivity, simplicity, and low-cost operation [244]. During compression molding, especially when carrying out the shape forming, process parameters such as temperature, time, and pressure are critical to the output of the material formed [278]. Typically, the temperature is maintained at 180–250 °C and time around 125–300 s while molding. Nevertheless, these temperature and time parameters are thought to be relatively long and energy-intensive [279]. Magnesium stearate and guar gum are widely used as release agents for the foams [280]. The densities (skeletal and bulk) of the released foams are strongly influenced by the type of starch. Glenn’s experiment obtained a density range of 0.12–0.15 g/cm^3^ [281]. There has been growing interest in finding other properties, such as the modulus of elasticity of starch-based foams, using compression molding [282].

### 9.3. Injection Molding

Injection molding (IM) is another preferred method for producing biofoams. Because of its distinct advantages, this technology is very widely used. An injection molding machine usually has numerous components that allow for distinct unit activities such as injection, molding, ejection, core pulling, and cooling. The screw chamber comprises a helical screw and a heating element, while the substrate (polymeric ingredients) is fed through a specifically built hopper. The starch-based polymer melts when heat is applied, and the screw homogenizes the ingredients, allowing for increased flowability. IM maximizes production rates without compromising strength, brittleness, shrinkage, and aesthetic qualities [283]. Furthermore, because the pressure of a gas and the resistance of fluid are directly related, cycle time and pressure in this process are substantially reduced. Several parameters such as nozzle speed, cooling time, and injection temperature may have a contributing effect on the output of microcellular foams [284]. The high setup cost, combined with the fact that only a low volume of the foam is fabricated, is the limitation of injection molding techniques.

### 9.4. Foaming

Foamed products are manufactured using physical, chemical, and mechanical techniques [285]. The difference between all the methods is significantly related to the materials and processes used. Water, argon, nitrogen, and carbon dioxide are commonly used as physical foaming agents because of their non-toxicity and other environmental advantages. The limitation of physical foaming agents is that some are fragile to provide needed mechanical strength. For instance, dextrin as a forming agent is more chemically stable; they are not better for high-strength products [37]. However, the mixture of foaming agents could provide both physical and mechanical characteristics, thus making them ideal for applications that demand a well-balanced material. Chemical foaming, on the other hand, involves the use of chemicals to trigger chemical reactions between the applied temperature, the gas, and the substrate. This method has been used for many decades’ past. Foaming agents used in this regard disintegrate at processing temperatures, releasing CO_2_ and nitrogen as a byproduct. Foaming agents from organic and inorganic compounds could be used for chemical foaming. However, for glass materials, chemical foaming agents and their advantages are CaSO_4_ (improves foaming ability of glass materials), carbonates, sulfates, and carbon black [286], while Na_2_HPO_4_ (reduces pore nonuniformity of foams) is used as a stabilizer [37].

Meanwhile, attaining better foaming efficiency is influenced by enthalpy reactions that produce the gas in the foaming process, which may be exothermic, endothermic, or a combination (exo-endothermic). Exothermic foaming agents have the most favorable foaming efficiency and are thus widely used in industrial processes. From an economic and efficiency viewpoint, chemical foaming agents offer superior advantages over physical foaming agents. Chemical foaming agents also provide consistent monitoring and nucleating effects, which can help alleviate moisture problems and improve mechanical characteristics. However, chemical agents react negatively by producing dangerous fumes when stored at higher temperatures.

## 10. Aerogels and Biofoams in Biomedical Fields

Aerogels and biofoams have unique appearances. These two are nanocellulose; however, they exhibit differing porosities. Biofoam is any multi-phase porous material with a porosity of more than 50%, in which gas (e.g., air) is distributed in a liquid, solid, or hydrogel. Pore size (or bubble diameter) is usually more than 50 nm. An aerogel is a mesoporous material with high porosity (>90%) with pore sizes ranging from 2 nm to 50 nm [287]. Biofoams have a heavier weight than aerogels. To the best of our knowledge, some agricultural wastes, mainly starch derived from protein-rich pulses, have not been widely used in the development of aerogels and biofoams. As a result, a component of this review focuses on industrial applications and manufacturing techniques for nanocellulose-based aerogels and foams. The research interest in nanocellulose-based aerogels and biofoams is relatively new, but it is growing swiftly [288]. Due to their ultralow density, configurable porous architecture, and outstanding mechanical capabilities, they are of interest for a wide range of applications, including biomedical scaffolds, thermal insulation, oil absorption, and flame retardancy [289]. Tissue engineering, drug delivery systems, surgical implants, biosensing, and disease detection and therapy are just a few of the applications where aerogels and biofoams have been researched in the biomedical field. According to research statistics, before 2016, 22% of all studies published on the application of nanocellulose materials, particularly aerogels, focused on drug delivery, 19% on tissue engineering, and 13% and 10%, respectively, on biomedical implant devices and antibacterial [290]. Various nanocellulose sources for drug delivery have been reported in various publications. Although chitosan, starch, and other nanocellulose sources are abundant in nature, their poor mechanical strength limits their use. Researchers’ efforts to resolve the setback have been impressive and encouraging. Radwan-Pragowska and colleagues created a pH-sensitive chitosan-based aerogel, using freeze-drying as a tool for cancer treatment [291]. Similarly, Martins et al. discovered that alginate-chitosan-based aerogels are non-toxic and have strong cell adhesion. Fundamentally, process parameters such as pH and nanocellulose content substantially impact the applicability of aerogels (e.g., pectin) [292].

Aerogels and biofoams are also used in restoring, maintaining, or repairing the biological function of wounded tissues or complete organs [293]. This idea comprises resurrection cells and tissues from their natural biological environment, then growing and multiplying them in vitro, using scaffold growth factors appropriate for the desired tissue. A three-dimensional porous scaffold is even more critical for housing cells and controlling their growth and regeneration [71]. Stem cells seeded in the nanocomposite showed significant bone differentiation, implying that they could be a good candidate for bone tissue regeneration scaffolding [294]. The influence of hyaluronic acid (HA) hydrogels on dental pulp stem cells’ behavior was reviewed, and findings from the study revealed that biocompatible and low immunogenic HA hydrogel scaffolds facilitated stem cell treatment in dentin/pulp injuries [295]. Nevertheless, certain disadvantages, such as quick in vivo breakdown and low mechanical strength, limit the use of HA hydrogels.

Aerogels and biofoams also have wound healing applications because they absorb large amounts of water while dry and donate water when hydrated [296]. Wound healing comprises a set of procedures designed to counteract the adverse effects of an injury’s biological reactions. The process involves immune cells (neutrophils, monocytes, macrophages, and lymphocytes), non-immune cells (endothelial, fibroblasts, and keratinocytes), soluble mediators (cytokines and growth factors), and extracellular matrix (ECM) components [297]. Wound healing or wound dressing could be categorized based on their components but, in most cases, are referred to as hydrocolloid dressings, hydrogel dressings, or alginate dressings [298]. Alginate, chitosan, collagen, and other biobased nanocellulose compounds have been employed in wound healing (Table 4). Alginate’s use became more widespread in the late twentieth century, and it has since become universal. Alginate is a natural biodegradable and biocompatible substance that can absorb 200–300 times its own weight in water [299]. Collagen, primarily found in bovine, porcine skin and tendons, bladder mucosa, and intestine, supports wound healing applications [300], and it is also used in various commercial products such as medical capsules and instrument strings [188]. We conclude that several similar biomolecules of therapeutic interest have received less attention than these well-studied and published biomedical applications of aerogels and biofoams. Advances will aid the commercialization of polymers with desired features in understanding the manufacturing process of biopolymers and their unique properties.

## 11. Knowledge Gaps

Aerogels and biofoams, developed from both renewable and non-renewable sources, are integral components of biomaterials. They have been deployed as a vital tool for diverse industrial uses such as food, medicine, and pharmaceuticals, making them potentially relevant and gaining widespread attention. Nonetheless, these materials are currently more popular in academia because a more significant percentage of literature surveys revealed that these biomaterials had been manufactured on a laboratory scale. As a result, non-biodegradable polymers (e.g., polyurethane and polyvinyl chloride) are dominant in many areas. Thankfully, the precursors (e.g., starch) for the manufacturing of aerogels and biofoams are commercially abundant. Our standpoint suggests that using organic precursors to develop biomaterials will mitigate the ineffective utilization of raw materials and fulfill the gaps within the circular economy concept geared towards the reuse and remanufacture of underutilized agricultural materials. Furthermore, the competitiveness of most biomaterials requires the scalability of all processes involved; thus, careful handling of the materials is crucial to its eventual marketability. After carefully reviewing the published articles, we, therefore, summarized our identified research gaps below:

1. Since biodegradability, biocompatibility, and sustainability are essential in conserving materials and ensuring their practical use, an in-depth survey of underutilized agricultural materials is essential to access their economic values.
2. Most literature did not characterize (e.g., purity) the raw starch used as a precursor for manufacturing biomaterials. Likewise, modified starch-based aerogel with unique mechanical properties and hydrophobicity could be a potential delivery system and food packaging option yet to be fully explored.
3. Most scale-up processes have associated limitations because of the processes and parameters involved. Therefore, critical evaluation unit operations such as drying and solvent exchange are crucial.
4. The modeling of a continuous process will improve the understanding of the process, since all available data in this research area are empirical-based.
5. Though there is considerable success in measuring the morphological and structural characteristics of biomaterials, most tests have not been standardized, and thus comparability of the values becomes difficult. In addition to available data on mechanical properties, there is a scanty survey of literature on the acoustic properties of biomaterials, even though biomaterials (aerogels and biofoams) are reported as a sound absorption material.

## 12. Perspective and Conclusions

Having surveyed the literature loop, we (the authors) are unarguably convinced that inorganic aerogels, and a few organic aerogels with resorcinol formaldehyde as a precursor, dominate the polymer sector because of their suitability for some engineering and non-engineering applications. However, because of their poor degradability and possibility of releasing harmful toxic substances, attention has now been shifted to complete natural biobased polymers such as starch. Notably, polysaccharides (alginate, cellulose, pectin, chitosan, chitin, and starch), lignin, proteins, and other biopolymers have been employed as natural precursors for biobased polymers. The findings of numerous academics revealed that they exhibited being microporous and mesoporous, having low densities, and having high surface areas, among other features. However, if laboratory cost comes with many routine processes, it is evident that the industrial price of these biomaterials will have associated exorbitant fees. Therefore, the pursuit of suitable raw materials with improved sustainability and lower pricing must continue, especially when biopolymers are desired for various applications. Similarly, the weak mechanical properties of natural precursors are one of the concerns of using them for biomaterial processes. Nevertheless, incorporating fillers provides a better framework, which is a panacea for their weak mechanical strength. On a molecular level, synergetic effects between distinct precursors and additions must be understood and predicted. In most cases, this is the only approach to creating novel hybrid materials rationally. Biomaterials have been used in pharmaceuticals (wound healing, drug delivery, etc.) and food (packaging). The expectation of these industries will be met if there is a reduction in the processes of these biomaterials. Therefore, the fundamental knowledge of the thermodynamics and kinetics of the solvent exchange and drying processes and their translation into multiscale models are essential to achieving this goal. We expect rising concerns of biomaterials (aerogels and biofoams) in the coming years. One of those concerns that might trigger scholars’ interest is how to reduce the processing time of all the stages involved in the manufacture of biomaterials. The retrogradation step in monolithic-manufactured aerogels cannot be a semi-continuous process, which usually necessitates a lengthy autoclaving time. The ability to directly produce aerogels in the form of particles or thin fibers, on the other hand, has a unique advantage: the solvent extraction time for small particles is faster than for monoliths, allowing for significant cost savings. Lastly, there is a considerable demand for biodegradable materials in numerous scientific fields. Unfortunately, while the synthetic porous materials have gained commercial acceptance, not minding their waste contribution, natural biobased materials are still seldomly transitioning from laboratory to industrial scale. Therefore, the development of scale-up techniques and novel process changes could mitigate this drawback. Furthermore, the research on the techno-economic analysis and life cycle assessment of aerogels and biofoams is minimal. As a result, we anticipate increased interest in these identified areas in the forthcoming years.

## Figures and Tables

**Table 1:** Extraction methodologies (wet milling) of starch sources.

Classfication	Substrates	Extraction Method	Autoclaving Temperature (°C)	Time (h)	References
Root and tuber	Purple Yam	Aqueous and alkaline	40–50	24	[68]
	Purple Yam	Aqueous and alkaline	120	0.5	[69]
	Potato	Aqueous and alkaline	37	5	[70]
	Cassava	Aqueous	55	24	[71]
	Cassava	Aqueous	50	24	[72]
	*Curcuma karnatakensis*	Aqueous and alkaline	-	-	[73]
	Purple flesh sweet potato	Aqueous and alkaline	50	12	[74]
Cereals and grains	Corn	Acid and alkaline	40	12	[75]
	Cooked corn	Aqueous and alkaline	-	-	[76]
	Rice cultivars	Aqueous and alkaline	-	-	[77]
	Rice cultivars	Alkaline steeping	20–22	-	[78]
	Soft and hard wheat	Aqueous and alkaline	40	48	[79]
	Wheat bran	Aqueous and alkaline	50	12	[80]
	Barley	Aqueous	-	-	[81]
	Rye grain	Alkaline and aqueous	20–22	-	[82]
	*Chenopodium album*	Alkaline and alcohol-aided alkaline	45	24	[83]
Pulses	Cowpea	Aqueous	40	48	[84]
	Mung bean	Aqueous	40	20	[85]
	Moth bean	Alkaline	40	24	[86]
	Faba bean	Alkaline	40	24	[87]
	Black bean	Alkaline and aqueous	40	12	[88]
	*Solanum lycocarpum*	Alkaline and aqueous	45	-	[89]

**Table 2:** Some selected starch modification methodologies.

Plant Source	Methodologies Used	Reagent	Outstanding Findings	References
Potato, maize, waxy maize, and high-amylose maize	Superheated steam (SHS)	Demineralized water	A potential benefit of SHS as a food ingredient is that a comparable product quality may be achieved at a lower caloric intake.	[110]
Potato, tapioca, corn, and wheat	Iterated syneresis	Water (temperature 90 °C, 4 h)	The starch crystallites of the modified starches melted at temperatures corresponding to those for retrograded starches, but the enthalpy change was evidenced to be higher.	[111]
Waxy maize (native and hydroxypropylated) and potato starches	Thermally-inhibited treatment	Distilled water (70 mL)	Waxy maize starches were heat-resistant. The potato was susceptible to heat. Control of reaction condition, dry heating process, and proper selection of ionic gum tends to improve starch functionality.	[112]
Potato	Osmotic pressure treatment (OPT) and heat-moisture treatment (HMT)	Saturated Na_2_SO_4_	The morphological characteristics of OPT starch granules changed into a folded structure after the treatment, whereas the HMT starch did not.	[113]
Potato	Multiple deep freezing and thawing	Liquid nitrogen	Granule surface has profound changes. The freezing/thawing process influenced the gelation characteristics, water solubility, and water holding capacity, while the branching characteristics of the starch granules remained unchanged.	[114]
Waxy maize	Instantaneous controlled pressure	Steam pressure	Increasing steaming time and temperature induced an increase in temperature transition (T_o_ and T_p_) and a reduction in gelatinization enthalpy.	[115]
Standard maize starch, waxy maize starch, and wheat starch	Drop (DIC) process	Saturated steam	The enzymatic susceptibility of starches was directly related to the structural modifications produced by the DIC treatment.	[115]
Cassava	Mechanical activation with stirring ball mill	Deionized water	Mechanical activation decreased the gelatinization temperature, enthalpy, apparent viscosity, and shear thinning of cassava starch and increased its cold-water solubility.	[116]
Cassava	Micronization is a vacuum ball mill	Deionized water	Easier gelatinization of micronized starch	[117]
Corn	Pulsed electric field treatment	Deionized water/KCL	PEF treatment led to an intragranular molecular rearrangement of corn starch granules.	[118]
Corn	Corona electric discharges	Water (25 °C)	CED treatment did not modify the behavior of corn starch during the freeze-thaw process	[119]
Potato, corn, and rice	Oxidation	Deionized water, NaOH, HCL, and NaOCL	The oxidation level directly affected the degree of crystallinity of starch and the degree of polymerization of amylose. The adhesion property of oxidized starch was mainly attributed to its granular size and shape.	[75]
Cassava, banana varieties, and corn	Esterification (acetylation, acylation, and phosphorylation)	STMP and STPP	Caused changes to some essential components such as thermal property and optical characteristics. Crystallization or retrogradation of starch granules was observed.	[120]
Cereals, root, and tubers	Crosslinking	-	Minimized granule rupture but decreased the solubility.	[121]
Cereal, root and tubers, legumes, and fruits	Graft polymerization	-	Changes in starch structure from a homopolymer to a heteropolymer.	[45]
Corn	Glucoamylase, bacterial α-amylase, fungal α-amylase, β-amylase, glucose isomerase, pullulanase, xylanase, and fungal acid protease RNA interference		Improvement of emulsification properties.	[122]

**Table 3:** Preparation methods for bioplastic starches.

Starch Source	TPS Polymer Blends	Plasticizer	Preparation Method	Conclusions	Research Gaps	References
Cassava	Starch/PCL	-	Extrusion	The TPS-PCL binary lines are largely implausible, but due to the chemical structure of the thermoplastic starch and the polyprolactone, they can be utilized to prevent the development of hydrogen boilers, which is both repercussive and unpredictable.	-	[248]
Corn	Starch/PCL	Corn starch, water, glycerol	Extrusion/compression molding	Gas and water vapor barrier characteristics were equivalent to synthetic polymers routinely used in food packaging.	The overall migration in various food simulants must be evaluated by the migration limits set by the regulation for plastic materials in contact with food.	[249]
Cassava	Starch/PBS	Glycerol	Extrusion	The addition of a maleic anhydride compatibilizer to PBS blends could provide strength and elongation at break to TPS/PBS blends significantly and improve interfacial miscibility.	-	[218]
Corn	Starch/PBAT	Glycerol	Extrusion (injection molding)	With increasing PBAT content, there is a clear difference between the mechanical and structural characteristics of compatibilized and non-compatibilized polymer blends.	The water intake of the blends is lower after compatibilization, and the duration to reach equilibrium water uptake is more significant than for non-compatibilized blends.	[250]
Corn	Starch/PLA	Glycerol	Extrusion	The combination of polymer blends significantly improved morphology and interfacial adhesion between the continuous starch phase 1:1 blend.	Increasing the thermoplastic continuous phase’s resistance to humidity absorption is necessary.	[251]
Corn and destructurized TPS	Starch/PBS	Glycerol, stearic acid	Extrusion	TPS-based compatibilizer enhanced TPS integration within the polyester matrix and increased tensile strength and tear resistance.	Investigation of the optimization of barrier properties of TPS-based film materials, as well as an examination of biodegradation mechanisms	[252]
Corn starch	TPS/PLA	Glycerol and water	Extrusion	Compared to virgin PLA, blending TPS with PLA resulted in a significant reduction in mechanical strength, indicating poor compatibility between the two materials.	Ongoing studies on investigating the effects of varied starch levels and increased compatibilizer concentrations.	[244]
Potato	Starch/PHBV, starch/PHB, starch/PLA	Glycerol	Extrusion/hot pressing	Fine morphology of starch remained in the PHB/EVA/starch blends.	-	[253]
Corn	Starch/PCL	-	Hot pressing	This type of blend is an intriguing approach to low-cost biodegradable materials, used, for example, to boost the usage of ecologically friendly materials in the packaging industry or to be utilized as fertilizer transporters in fertilizer control releases.	Due to their differing polarity, the lack of adhesion between the polysaccharide and synthetic polymer matrices is the fundamental drawback of the starch/PCL mixes.	[254]
Corn	Starch/PLA	Glycerol	Hot pressing	In the continuous starch phase, the PLA dispersion was better in the starch-PLA matrices compatible with grafted PCL, notably for the maximum amount of compatibilizer.	-	[255]
Corn	Starch/PLA	Glycerol	Compression molding	Compatibilizer did not affect biodegradability but caused a positive deviation from the mixture rule for the blend samples’ tensile characteristics, indicating a good compatibilization efficiency.	-	[245]
Corn	Starch/PCL	-	Cold pressing	The intrinsic biodegradability is influenced more by the compatibilization efficiency than by the starch concentration in the polyester matrix.	-	[256]
Tapioca	Starch/PVA	Acetyl tributyl citrate (ATBC), glycerol	Extrusion blowing	In the starch/PVA/OMMT system, the mixed plasticizers (ATBC and GLY) produced highly stable and stronger hydrogen bonds.	Hydrophobic plasticizers should be investigated further to increase the dispersion of OMMT and the performance of starch-based nanocomposites.	[257]
Corn	Starch/PVA/CNT	Glycerol	Solution casting	The addition of CNT improved the compatibility of PVA/starch blends, according to thermal stability, water uptake, and microscopic studies.	Loss of tensile strength, modulus, and elongation at break is caused by incorporating starch into the PVA matrix.	[258]
Maize	Starch/PVA/films	Water	Solution casting	All the films are biodegradable and present good antioxidant properties compared to the standard sample. Increasing the 7H4MC content in the blend matrix enhances the antioxidant property.	Further investigation is needed to ascertain the reasons behind the reduction in the water vapor transmission rate (WVTR) of films containing 7H4MC concerning the thickness of films.	[259]
Wheat	Starch/PLA	Glycerol	Extrusion	PLA addition to TPS caused a transformation into brittle materials	Investigation of the copolymer or reactive blending is necessary to overcome the difficulty of respective constituents.	[260]
Sugar palm	Starch/PLA	Cellulose, glycerol, sorbitol	Solution casting	As the TPS loading increased, the density, water absorption, and thickness swelling increased, linked to the significant functional group of hydroxyls.	The potential of TPS/PLA in food packaging as a biodegradable material should be further demonstrated.	[261]
Cassava	Starch/PET					[262]
Maize	Starch/PLA		Extrusion/injection molding	Blending PLA with TPS is a cost-effective and ecologically responsible technique to increase the hardness and ductility of PLA, allowing it to be used in more applications.	Poor miscibility between TPS and PLA.	[263]
Cassava	Starch/PLA	Glycerol	Extrusion	The DA of acetylated starch had an impact on the PLA/TPSA mix films’ morphologies and their performance.	The PLA/TPSA blend film showed noticeable phase separation, resulting in worse characteristics.	[264]
Cassava	Starch/PLA	Glycerol	Cast fil extrusion/Compression molding	The use of MA as a compatibilizer increased the interfacial adhesion between PLA and TPCS, with the effect being more significant in blends made with DCP rather than L101 as the initiator.	The properties of reactive blend films are not the same as those of industrial-scale processes.	[265]
Wheat	Starch/PLA	Sorbitol, glycerol		Plasticizer transfer to the matrix results in decreased tensile strength and modulus in the solid-state but a higher crystallization rate upon heating due to increased chain mobility.	-	[266]
Corn	Starch/PLA	Sorbitol, glycerol	Extrusion	Rheological tests demonstrated a link between the storage modulus and complex viscosity of PLA/TPS blends and their morphology, i.e., the rheological behavior of the polymer blend with matrix dispersion morphology is more influenced by the matrix phase.	-	[267]
Corn	WF/Starch/PLA	Glycerol	Extrusion	The water resistance of the blends decreased as the starch/WF ratio decreased.	-	[268]

**Table 4:** Biomedical applications of nanocellulose materials and processing methods.

Potential Application	Nanocellulose Sources	Preparation Method	Drying Methods	Summary of Essential Observations and Drawn Conclusions	References
Drug delivery/carrier	Chitosan	Sol-gel	ScCO_2_	Gel shrinkage throughout the ScCO_2_ drying process. Temperature and pH in the aqueous media can degrade the aggregate structure of chitosan and change the pore structure of chitosan aerogels.	[291,301]
	Alginate	An aqueous solution of sodium alginate	ScCO_2_	Drugs loaded in alginate-based aerogel particles are primarily amorphous.	[302]
	Protein	Sol-gel	FD, ScCO_2_	Facilitating the structural analysis of biological systems is best achieved using supercritical drying. The drug-loaded whey protein aerogels showed a sustained drug release at gastric (pH 1.2) and intestinal (pH 6.8) simulated digestive pH conditions.	[303]
	Cellulose	Solvent exchange	ScCO_2_	Solvent exchange scCO_2_ impregnation proved an effective single-step procedure for drug loading and aerogel formation. In addition, due to their high in vitro biocompatibility, cellulose aerogel micro fibers showed immediate drug release behavior.	[304]
	Pectin	Diffusion and internal setting	ScCO_2_	Spherical and monolithic pectin aerogels, which combine pectin and aerogel properties, show promise as very porous drug carriers with highly selective surface areas capable of controlling drug release.	[305]
	Gelatin	Sol-gel	ScCO_2_	The rapid desorption and dissolution of the pharmaceuticals from the loaded aerogel is aided by rich hydration of the silica gelatin skeleton, according to simultaneous analysis of all relevant kinetic and structural data.	[306,307]
Tissue engineering	Alginate lignin	Sol-gel	ScCO_2_	Alginate–lignin aerogels were found to be non-cytotoxic and to have strong cell adhesion in cell tests, making them promising candidates for various applications such as tissue engineering and regenerative medicine.	[308]
	Chitosan	Sol-gel	ScCO_2_, FD	ScCO_2_ drying produced a smaller particle size, and the technology created salbutamol-loaded chitosan aerogel microparticles that could be used in pulmonary medication delivery systems.	[309]
	Alginate-collagen	Water-solvent	ScCO_2_	Stable aerogel is a crucial indicator of cell adhesion and proliferation in the collagen-alginate-GO aerogel-based scaffold.	[310]
	Chitin-hydroxyapatite composite (ChHA)	Sol-gel	ScCO_2_	The chitin-hydroxyapatite (ChHA) composite was well distributed within the composite structures. ChHA matrices could be used in bone tissue engineering.	[311]
	Cellulose nanofibers (CNF)/chitosan	Sol-gel	Lyophilization	Compared to pure-CNFs and pure-CS aerogels, CNFs/CS aerogels offer superior characteristics, and the manufacturing of CNFs/CS aerogels is promising for tissue engineering applications.	[312]
Biomedical implantable devices	Polyurea silica aerogel (PCSA)	Sol-gel	ScCO_2_	There was no evidence of harm in the tissues surrounding the implants or in the distant organs of rats. The implants did not show any visible or noteworthy alterations in any location. Only age-related alterations were discovered after a thorough necropsy and tissue histology (Sabri).	[290]
Wound care/healing	Cellulose (nanocrystalline cellulose and nanocellulose aerogel	-	-	Peptide loading, surface charge, and protease sequestration were higher in the nanocellulose materials (pNA and pNC) than in cellulosic filter paper (CFP). Nanocellulose materials are promising biosensor transducer surfaces.	[313,314]
	Nanocellulose/nanocarbon composites	-	-	Nanocellulose/carbon nanotube composites positively impact the adhesion and development of human and swine adipose tissue-derived stem cells, mainly when grown in a pressure-generating lab-made bioreactor.	[315]
	Alginate	Internal setting gelation and solvent exchange	ScCO_2_	Alginate aerogels (Ca-Zn-Ag) demonstrated excellent liquid absorption and high liquid retention capabilities in any formulae.	[316]
	Collagen	Sol-gel	Lyophilization	Functionalizing nutraceuticals on collagen can result in very stiff and porous aerogels with bio-functional properties and significant biocompatible capabilities for regulated drug administration in cell and tissue regenerative applications.	[317]
	Chitosan-alginate	Sol-gel	ScCO_2_	Cell-based experiments demonstrated the non-cytotoxicity and bioactivity of the aerogels, thus hastening wound closure in an in vitro model of cell monolayer recovery.	[318]

## Data Availability

Not applicable.
